# Role of Scl39a13/ZIP13 in cardiovascular homeostasis

**DOI:** 10.1371/journal.pone.0276452

**Published:** 2022-10-21

**Authors:** Takafumi Hara, Ikuko Yamada, Takuto Ohashi, Masaru Tamura, Atsushi Hijikata, Takashi Watanabe, Minghao Gao, Kana Ito, Saeko Kawamata, Shiori Azuma, Emi Yoshigai, Yukiko Sumiyoshi, Natsumi Yasuhiro, Osamu Ohara, Heloísa G. dos Santos, Toshiyuki Fukada

**Affiliations:** 1 Molecular and Cellular Pathology, Faculty of Pharmaceutical Sciences, Tokushima Bunri University, Yamashiro-cho, Tokushima, Tokushima, Japan; 2 Technology and Development Team for Mouse Phenotype Analysis, BioResource Research Center, Riken, Japan; 3 Faculty of Bioscience, Nagahama Institute of Bio-Science and Technology, Nagahama, Shiga, Japan; 4 Laboratory for Integrative Genomics, RIKEN Center for Integrative Medical Sciences, Kanagawa, Japan; 5 Research Fellow of the Japan Society for the Promotion of Science (JSPS), Japan; 6 Kazusa DNA Research Institute, Chiba, Japan; 7 Medical Genetics Service S. Maria Hospital, Lisbon, Portugal; The Ohio State University, UNITED STATES

## Abstract

Zinc plays a critical role in many physiological processes, and disruption of zinc homeostasis induces various disorders, such as growth retardation, osteopenia, immune deficiency, and inflammation. However, how the imbalance in zinc homeostasis leads to heart disease is not yet fully understood. Cardiovascular diseases are a major cause of death worldwide, and the development of novel therapeutic targets to treat it is urgently needed. We report that a zinc transporter, ZIP13, regulates cardiovascular homeostasis. We found that the expression level of *Zip13* mRNA was diminished in both primary neonatal cardiomyocytes and mouse heart tissues treated with the cardiotoxic agent doxycycline. Primary neonatal cardiomyocytes from *Zip13* gene-knockout (KO) mice exhibited abnormal irregular arrhythmic beating. RNA-seq analysis identified 606 differentially expressed genes in *Zip13*-KO mouse-derived primary neonatal cardiomyocytes and Gene ontology (GO) analysis revealed that both inflammation- and cell adhesion-related genes were significantly enriched. In addition, telemetry echocardiography analysis suggested that arrhythmias were likely to occur in *Zip13*-KO mice, in which elevated levels of the cardiac fibrosis marker *Col1a1*, vascular inflammation-related gene *eNOS*, and Golgi-related molecule *GM130* were observed. These results indicate the physiological importance of ZIP13—it maintains cardiovascular homeostasis by resolving inflammation and stress response. Our findings suggest that optimizing ZIP13 expression and/or function may improve cardiovascular disease management.

## Introduction

Despite recent progress in clinical research and therapy, cardiovascular diseases (CVDs) represent a major cause of mortality and morbidity worldwide [1]. The hallmarks of CVDs include cardiac dysfunction, cardiomyocyte apoptosis, fibrosis, hyperplasia, inflammation, and ventricular remodeling. Both genetic and environmental factors pathologically contribute to the development of CVDs; however, the precise causes that trigger cardiac diseases and their progression remain largely unknown [2]. Hence, it is important to establish new therapeutic approaches to reduce morbidity and mortality associated with heart failure.

Zinc serves as a structural component of many proteins, including enzymes, transcription factors, and signaling molecules. It is obtained by the human body through food intake, absorbed in the intestine, and distributed to tissues through the circulatory system [3]. Zinc deficiency can lead to stunted cell growth and serious metabolic disorders, whereas excess zinc can be cytotoxic. Mammalian cells have evolved a complex zinc transport network to maintain intracellular zinc homeostasis. Two families of transporters, ZIPs (Zrt/Irt-like protein, SLC39A) and ZnTs (SLC30A), regulate the influx, efflux, and intracellular compartmentalization of zinc ions. These are involved in a variety of mammalian physiological processes and may also contribute to the development of diseases in humans [3, 4].

ZIP13/SLC39A13, a member of the SLC39A/ZIP family, plays an important role in the development of connective tissue in mice and humans [5, 6]. Loss of function mutations in ZIP13 lead to growth retardation, skeletal dysplasia, relatively short stature, hyperplastic skin, and skeletal dysplasias. ZIP13 dysfunction contributes to the development of Ehlers–Danlos syndrome spondylocheirodysplastic type 3 (EDSSPD3, OMIM 612350), a very rare autosomal recessive disease. ZIP13 is required for normal cardiac function as it modulates calcium/calmodulin-protein kinase II (CaMKII) signaling in cardiomyocytes [7]; hence, ZIP13-mediated zinc homeostasis in cardiomyocytes may affect cardiovascular physiology and pathophysiology *in vivo*. However, there is limited information regarding the physiological functions of ZIP13 in cardiovascular homeostasis.

In the present study, we evaluated doxorubicin (Dox)-induced CVD models using *Zip13*-deficient (KO) mice and demonstrated that ZIP13 plays a role in cardiovascular homeostasis by resolving inflammation and stress responses. Our findings suggest that ZIP13 significantly affects cardiac physiology; therefore, it is a potential therapeutic target for CVD treatment.

## Materials and methods

### Animal experimentation and care

*Zip13*-KO mice were developed using the techniques developed previously [6]. All mice were housed at a constant room temperature of 22°C, under 12 h light/dark cycle, and fed either a normal or a powder chow diet for WT and *Zip13*-KO mice, respectively, with water *ad libitum*. All experimental procedures were approved and in accordance with the guidelines of the Animal Facility Center of Tokushima Bunri University and the Institutional Animal Care and Use Committee of RIKEN Tsukuba Branch.

### Primary neonatal cardiomyocytes (PNCs) culture

PNCs were prepared from newborn c57BL/6N-or *Zip13*-KO mice using a primary cardiomyocyte isolation kit (Thermo Scientific Co., Ltd., MA, USA). Post-natal day-3 heart tissues were minced and dissociated using an enzyme solution containing papain and thermolysin. The cells were cultured at a density of 5.0×10^5^/well in a 24-well plate with Dulbecco’s modified Eagle’s complete medium with growth supplements (provided in the kit), 10% fetal bovine serum (Gibco Invitrogen, CA), and 100 U/ml penicillin/0.1 mg/ml streptomycin (Sigma-Aldrich) in a humidified incubator at 37°C with 5% CO_2_. The medium was replaced every other day. Cardiomyocyte beating was observed after 3–4 d of culture.

### Beating analysis of PNCs

The beating of PNCs was analyzed using ImageJ software. Beating was observed and recorded as movies of phase-contrast microscopic images using a Keyence BZX-810 microscope (Keyence Co., Ltd., Japan) with a 10x objective (Nikon Plan APO 10x/0.45), and the recorded data were imported to ImageJ software as stack images in grayscale. Time-dependent changes according to the brightness of pixels at the edge of the cardiomyocytes were quantified and expressed as kymographic data.

### Telemetry echocardiography (ECG) recording and analysis

Telemetry ECG recordings were conducted after the surgical implantation of wireless radiofrequency telemetry devices (ETA-F10; Data Sciences International (DSI), St Paul, MN, USA) in the intraperitoneal space under general anesthesia with a mixture of 0.5 mg/kg body weight (b.w.) medetomidine, 4.0 mg/kg b.w. midazolam, and 5.0 mg/kg b.w. butorphanol. After the surgery, atipamezole 0.5 mg/kg b.w. was administered subcutaneously to the neck as an antagonist. Two weeks after the devices were implanted, telemetry ECG recording was performed for 24 h. Data was recorded again two weeks after a single intraperitoneal injection of 15 mg/kg of doxorubicin (Dox; Combi-blocks Inc., CA, USA).

### RNA isolation, cDNA synthesis, and PCR analysis

Total RNA was isolated from cells and heart tissues of mice using Sepasol (Nacalai, Japan), and cDNA synthesis was performed using the PrimeScript RT Reagent Kit (Takara Bio, Japan). Real-time quantitative RT-PCR analysis was performed using SYBR Green qPCR reagent (TOYOBO, Japan) with gene-specific primers and a QuantStudio3 Applied Biosystems instrument (Thermo Fisher, TX, USA).

### Gene-expression analysis using mRNA-seq analysis

Total RNA was extracted from PNCs of either wild-type (WT) littermates or *Zip13*-KO mice using Sepazol, and total RNA samples were subjected to RNA sequence analysis using Illumina HiSeq 1500 [8]. The reads from RNA sequencing were aligned to the mouse reference genome (GRCm38) using HISAT2 [9], and the expression level of each gene was quantified as count per million mapped reads (CPM) using StringTie2 [10]. Differentially expressed genes with FDR (false discovery rate) < 0.05, and fold-change > 2.0, between WT and *Zip13*-KO cells (WT vs. *Zip13*-KO) were selected using EdgeR software [11]. The differentially expressed genes were subjected to a functional enrichment analysis algorithm [12] implemented in an in-house Python program, and the gene ontology (GO) biological process terms with FDR<0.05 were considered to be significantly enriched in the upregulated or downregulated genes.

### Histology

Hematoxylin and eosin (H&E) staining was used to examine histological alterations in heart tissue. Briefly, steady-state heart tissues preserved in 4% paraformaldehyde were dehydrated and paraffin-embedded sections were prepared. After H&E staining, the heart histomorphology was observed. For immunohistochemistry (IHC) analysis, anti-TGN46 (Novus Co., CO.) and anti-laminin-2α (Sigma-Aldrich) antibodies were used in frozen sections of the heart tissues, followed by staining with Alexa Fluor 594-conjugated goat-anti-rabbit IgG and Alexa Fluor 488-conjugated goat-anti-mouse IgG antibodies (Life Technologies), respectively. The sections were mounted using Vectashield with DAPI (4′,6-diamidino-2-phenylindole) (Vector Labs, CA, USA).

### Statistical analysis

The results are represented as the mean ± S. D. Differences amongst the groups were analyzed using Student’s *t*-test. Statistical significance was set at P < 0.05. Data from the telemetry ECG analysis were statistically analyzed by two-way analysis of variance (ANOVA) to estimate the interactive effects of the *Zip13* gene and Dox injection on cardiovascular functions.

### Case reports

We were notified of the following clinical information regarding EDSSPD3 patients (siblings) that was not documented in the previous publication [6]:

- at 22 years of age, the affected brother suffered cerebral hemorrhage in the left putamen;
- at 26 years of age, the affected sister suffered from a subarachnoid hemorrhage after an atherothrombotic brain infarction (ischemia) of the left cerebral arteria media secondary to a probable dissection of the intracranial portion of the left internal carotid artery;
- Normal echocardiogram in both subjects at this moment;
- the mother of the siblings, who possesses the heterozygotic *ZIP13/SCL39A13* allele with a pathogenic mutation, had experienced arrhythmia, although the connection between her heterozygotic *ZIP13* allele and arrhythmia is unclear.

## Results

### Effect of cardiotoxic stress on *Zip13* gene expression in cardiomyocytes

**Fig 1:**
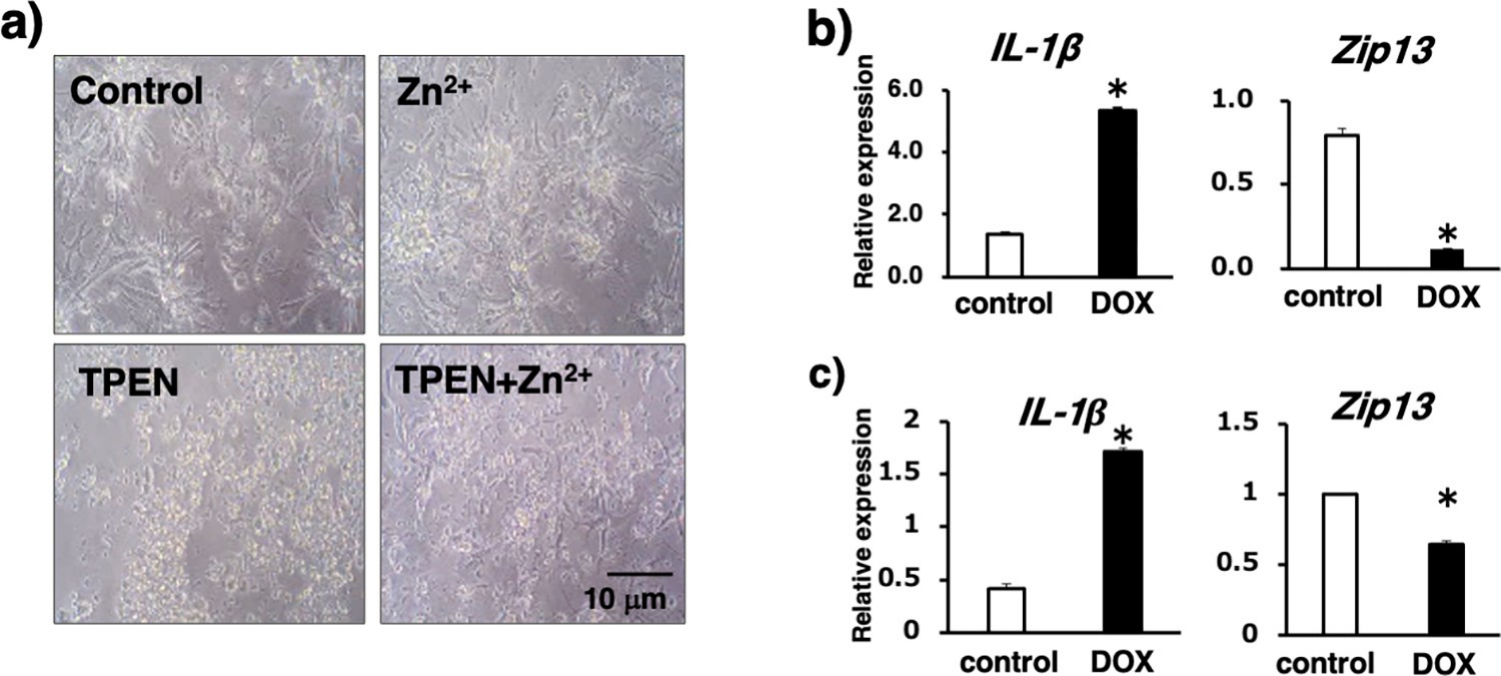
Effects of zinc level alteration and Dox stimulation on PNCs. **(a)** Effect of either zinc depletion or supplementation on the morphology of PNCs cultured in media containing 10 μM TPEN or 100 μM ZnSO_4_ for 24 h. Representative images are shown (n = 3–4; scale bar = 10 μm). **(b)** Effect of Dox treatment on *Il-1β* and *Zip13* expression in PNCs. PNCs were cultured with or without Dox (10 ng/mL) for 24 h. **(c)** Effect of Dox treatment on *Il-1β* and *Zip13* expression levels in heart tissues of mice. C57BL/6J mice were intraperitoneally injected with Dox (15 mg/kg), and heart tissue samples were collected two weeks after Dox injection. All data were collected from three to four independent experiments. Significant differences were analyzed using the Student’s *t*-test for each experimental group (* p < 0.05).

The physiological effects of zinc as an essential trace mineral on cardiovascular homeostasis have been reported in both experimental and clinical reports [13–16]. However, the role of zinc ion in cardiomyocytes remain unclear. Therefore, we examined the effects of zinc on morphological changes in murine PNCs. Supplementation with 100 μM zinc did not cause any apparent morphological changes in PNCs (Fig 1A, upper right). Conversely, supplementation with 10 mM of the cytosolic zinc chelator TPEN [17] induced remarkable cellular alterations, such as shrinking of cell shape, with some cells detaching and floating freely in the culture dish (Fig 1A, lower left). These abnormal phenomena were reversed following the addition of zinc (Fig 1A, lower right), indicating that cytosolic zinc is essential for the cellular physiology of PNCs and demonstrated a critical role in their survival.

The thoracic aortas of *Zip13*-KO mice [18] are notably fragile. Hence, we evaluated whether ZIP13 affects cardiovascular function by first examining whether the expression level of ZIP13 was affected by cardiotoxic Dox. Dox was selected as a clinical anticancer drug because it is known to induce cardiotoxicity, which is one of the major adverse effects leading to arrhythmia, heart failure, and hypotension [19]. Dox treatment increased *IL-1β* mRNA expression level in PNCs, mimicking inflammatory conditions (Fig 1B, left), whereas *Zip13* expression level significantly decreased (Fig 1B, right). Similar to this *in vitro* result, peritoneal injection of Dox also upregulated *IL-1β* and simultaneously decreased *Zip13* expression level in heart tissues (Fig 1C). These results indicate that ZIP13 is sensitive to certain types of cardiotoxic stress and its loss of function may be a factor in some clinical conditions caused by CVDs.

### Loss of ZIP13 impaired cardiomyocyte function

**Fig 2:**
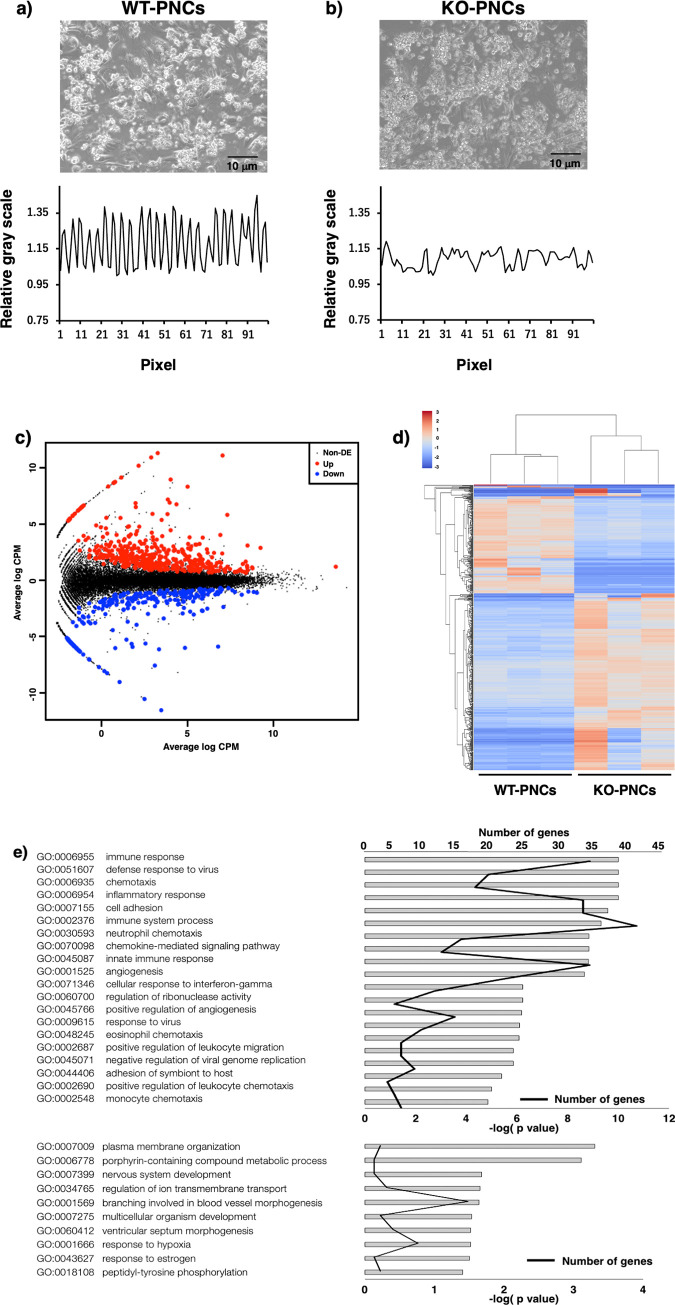
ZIP13-deficient PNCs exhibited abnormal morphology and impaired beating rhythm. **(a)** Morphological (top) and beating (bottom) of PNCs in WT mice. PNCs were cultured as described in the Materials and Methods. The heartbeats of cardiomyocytes were recorded under a bright field of microscopic observation as a movie file. The data were analyzed using ImageJ software. Beating was quantified as grayscale color change at the edge of the cardiomyocytes. **(b)** Morphological and beating observations of PNCs in *Zip13*-KO mice. Aberrant shrinking morphology (upper) and irregular beating (lower) were observed. Representative images are shown (n = 3–4; scale bars = 10 μm). **(c)** MA-plot of RNA-seq data of WT- and *Zip13*-KO-derived PNCs samples. The obtained total RNA was subjected to RNA-seq analysis. **(d)** Heat map analysis of the CPM of 607 genes in WT and *Zip13*-KO derived samples. **(e)** GO terms enriched in genes upregulated in PNC samples of *Zip13*-KO mice. The grey bar represents the log (p-value) of each GO term. The black lines indicate the number of genes. GO terms enriched in downregulated genes were downregulated in the *Zip13*-KO samples.

We next determined whether the loss of ZIP13 function is directly associated with cardiomyocyte characteristics. Compared with PNCs derived from WT mice (WT-PNCs, Fig 2A upper and S1 Video), those from *Zip13*-KO mice (KO-PNCs) showed aberrant shrinking morphology (KO-PNCs, Fig 2B upper and S2 Video). The KO-PNCs also exhibited irregular beating (Fig 2B lower and S2 Video), unlike the WT-PNCs with constant rhythms (Fig 2A lower and S1 Video). We then performed RNA sequencing followed by MA plot analysis using WT- and KO-PNCs to define and characterize the roles of ZIP13 in PNCs and found that 392 genes and 214 genes were up- or down-regulated in KO-PNCs, respectively (Fig 2C). These 606 genes were categorized into several major groups by heat map analysis using the CPM values (Fig 2D). Further GO term analysis emphasized that the genes related to “chemotaxis,” “inflammatory response,” and “cell adhesion” exhibited increased expression levels in KO-PNCs; moreover, the expression levels of genes associated with “membrane organization,” “regulation of ion transmembrane transport” and “ventricular septum morphogenesis” were diminished by the loss of ZIP13 (Fig 2E), indicating that ZIP13 is indispensable for the normal functions of cardiomyocytes, and that its deficiency may have profound effects on the physiology of cardiomyocytes leading to disturbance of the delicate balance that normally regulates cardiovascular homeostasis.

### *Zip13*-KO mice spontaneously exhibited arrhythmic features

**Fig 3:**
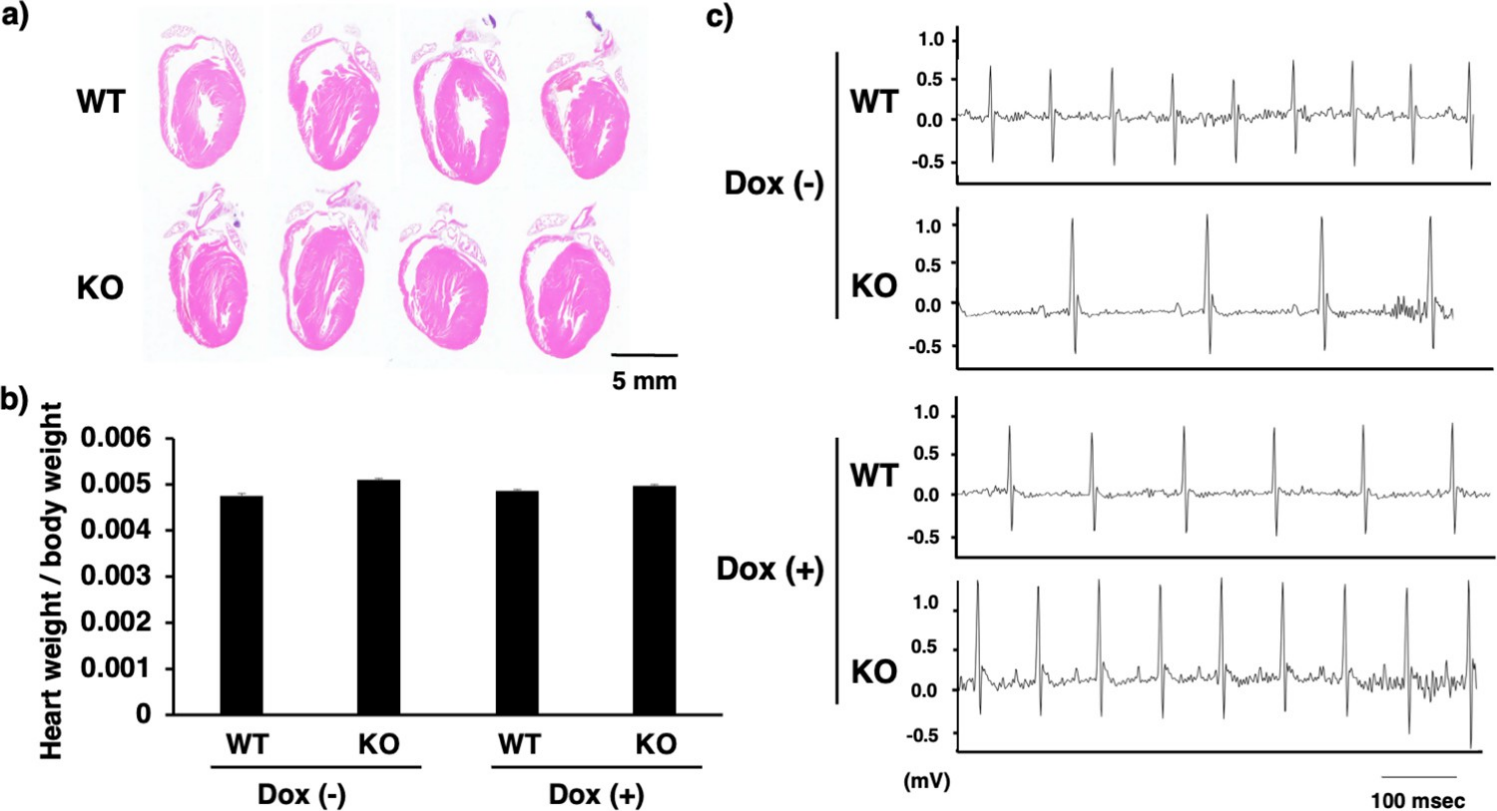
Cardiac analysis of *Zip13*-KO mice *in vivo*. **(a)** Histological analysis of steady-state heart tissue from WT and *Zip13*-KO mice. Heart tissue samples were sectioned longitudinally and stained with hematoxylin and eosin (n = 4). **(b)** Heart tissue weights of WT and *Zip13*-KO mice injected with or without Dox (normalized to body weight). n = 3 to 4. **(c)** Representative heartbeat analysis in either WT or *Zip13*-KO mice injected with or without Dox was monitored using the telemetry ECG system, as described in Materials and Methods. n = 3 to 4. Scale bar 100 ms.

Next, we examined the cardiac function of the *Zip13*-KO mice using histological and telemetry ECG analyses. We used a doxorubicin-induced cardiovascular disease model to clarify the involvement of ZIP13 in cardiac functions *in vivo*. There were no significant histological changes in the hearts of WT and *Zip13*-KO mice (Fig 3A), and the heart tissue weights normalized with the body weights also did not change in WT and *Zip13*-KO mice with or without Dox (Fig 3B). In contrast, the *Zip13*-KO mice spontaneously exhibited arrhythmic features without Dox injection (Fig 3C, upper panel). While Dox injection induced arrhythmic features (Fig 3C, lower), ZIP13 deficiency caused a significant increase in the QT, JT, and Tpeak intervals (Table 1), suggesting that ZIP13 may function as a key molecule that fine-tunes cardiac functions by resolving cardiovascular stresses. Two-way ANOVA analysis based on the results of telemetry ECG analysis indicated that the genotype and the Dox injection independently influenced cardiovascular parameters such as QT interval, QTc, JT interval, and Tpeak tend interval, in which the interactive effects of the genotype and Dox injection on cardiovascular parameters were not confirmed (Table 2), which clearly suggests that the spontaneous cardiac dysfunction observed in *Zip13*-KO mice occurred without relevant association with Dox administration, showing that ZIP13 plays an indispensable role in normal cardiac functions *in vivo*.

**Table 1:** Summary of telemetry ECG analysis of WT and *Zip13*-KO mice.

	Dox (-)		Dox (+)	
Parameters	WT	*Zip13*-KO	WT	*Zip13*-KO
RR Interval (s)	0.12 ± 0.0024	0.13 ± 0.0081	0.12 ± 0.0030^§^	0.12 ± 0.0069
Heart Rate (BPM)	492.87 ± 8.84	484.44 ± 23.73	531.75 ± 12.47^§^	533.20 ± 26.77
PR Interval (s)	0.0036 ± 0.00073	0.037 ± 0.0017	0.036 ± 0.00072	0.036 ± 0.0013
P Duration (s)	0.0021 ± 0.00094	0.015 ± 0.0025*	0.018 ± 0.0015	0.016 ± 0.00092
QRS Interval (s)	0.010 ± 0. 00022	0.0094 ± 0.00016	0.0096 ± 0.00025	0.0093 ± 0.00010
QT Interval (s)	0.0017 ± 0. 00031	0.019 ± 0.0015	0.020 ± 0.00047^§^	0.021 ± 0.00077
QTc (s)	0.049 ± 0.0012	0.053 ± 0.0011	0.058 ± 0.0014^§^	0.061 ± 0.00034^§^
JT Interval (s)	0.0071 ± 0.00018	0.0092 ± 0.0012*	0.0098 ± 0.00048^§^	0.011 ± 0.00063
Tpeak Tend Interval (s)	0.0049 ± 0.00018	0.0067 ± 0.0011*	0.0069 ± 0.00049^§^	0.0078 ± 0.00069
P Amplitude (mV)	0.0058 ± 0.0066	0.075 ± 0.022	0.047 ± 0.0059	0.068 ± 0.016
Q Amplitude (mV)	-0.0071 ± 0.0076	-0.0077 ± 0.00065	-0.0053 ± 0.0056	-0.00040 ± 0.0055
R Amplitude (mV)	0.68 ± 0.10	0.84 ± 0.25	0.67 ± 0.10	0.82 ± 0.18
S Amplitude (mV)	-0.29 ± 0.043	-0.39 ± 0.12	-0.26 ± 0.050	-0.35 ± 0.075
ST Height (mV)	0.038 ± 0.010	0.043 ± 0.018	0.047 ± 0.010	0.058 ± 0.015
T Amplitude (mV)	0.079 ± 0.015	0.094 ± 0.047	0.073 ± 0.020	0.079 ± 0.025

**Table 2:** Summary of results of two-way ANOVA of telemetry ECG analysis for WT and *Zip13*-KO mice.

Parameters	Genotype	Dox	Interaction
RR Interval (s)	ns	↓	ns
Heart Rate (BPM)	ns	↑	ns
PR Interval (s)	ns	ns	ns
P Duration (s)	↓	ns	ns
QRS Interval (s)	ns	ns	ns
QT Interval (s)	↑	↑↑	ns
QTc (s)	↑	↑↑↑	ns
JT Interval (s)	↑↑	↑↑↑	ns
Tpeak Tend Interval (s)	↑	↑↑	ns
P Amplitude (mV)	ns	ns	ns
Q Amplitude (mV)	ns	ns	ns
R Amplitude (mV)	ns	ns	ns
S Amplitude (mV)	ns	ns	ns
ST Height (mV)	ns	ns	ns
T Amplitude (mV)	ns	ns	ns

Results of the telemetry ECG analysis of WT and *Zip13*-KO mice with or without intraperitoneal injection of Dox were quantified, and the cardiovascular parameters are summarized in Table 1. Data are presented as the mean ± SE. Statistical analysis was conducted using Student’s *t*-test. *p < 0.05, compared between WT and *Zip13*-KO mice. ^§^p < 0.05, comparison between Dox (−) and Dox (+) of the same genotype.

We quantified the results of telemetry ECG analysis of WT and *Zip13*-KO mice with or without intraperitoneal injection of Dox. Two-way ANOVA with Bonferroni post hoc analysis was applied to examine the influence of genotype (genotype), Dox injection (Dox), and the interactions between genotype and Dox injection (interaction) on cardiac parameters. Data collected on the interactions between genotype and Dox treatment indicated the influence of genotype on Dox-induced cardiac functional changes and vice versa. The upper and lower arrows represent the statistically significant increase (↑) or decrease (↓) in the cardiac parameter values of *Zip13*-KO mice compared to those of WT mice, respectively. ↑, ↓: p < 0.05, ↑ ↑, ↓ ↓: p < 0.01, ↑ ↑ ↑, ↓ ↓ ↓: p < 0.001, ns: not significant.

### Gene expression profiling of heart tissues from *Zip13*-KO mice

**Fig 4:**
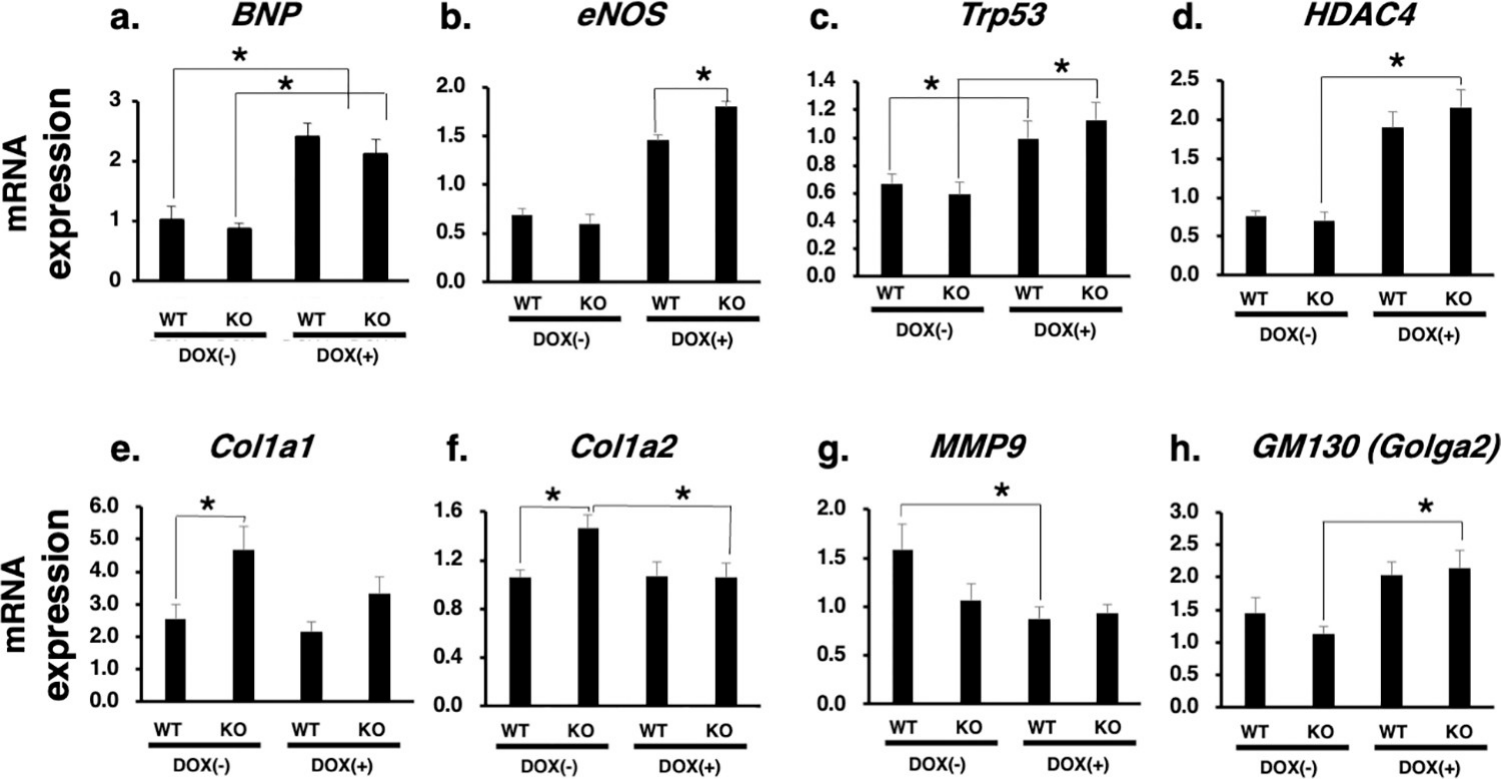
Gene expression profiling of *Zip13*-KO mice heart tissues. The expression of genes related to cardiomyocyte function (a: *BNP*; b: *eNOS*, c: *Trp53*, d: *HDAC4*, e: *Col1a1*, f: *Col1a2*, g: *MMP9*, and h: *GM130*) was analyzed in heart tissue samples derived from WT and *Zip13*-KO mice, with or without Dox administration. Each gene was quantitatively analyzed using qPCR. Significant differences were analyzed using the Student’s t-test, *p < 0.05, n = 3–4.

To clarify the correlation between telemetry ECG analysis and gene expression profiles, we assessed gene expression of representative cardiac molecules in the hearts of WT and *Zip13*-KO mice. Brain natriuretic peptide (BNP, Fig 4A) and endothelial nitric oxide synthase (eNOS, Fig 4B), both of which are known to be elevated in response to cardiotoxic stimuli, were upregulated in WT and *Zip13*-KO mice with Dox injection. They also increased *Trp53* expression level (Trp53, Fig 4C) by Dox injection, which has a protective effect against cardiotoxicity [20], indicating that the cardiac disease model induced by Dox injection was clearly developed in both mice. The expression pattern of histone deacetylase 4 (HDAC4, Fig 4D) demonstrated a trend similar to the three molecules above, suggesting that transcriptional and/or epigenetic alteration by Dox administration may influence gene expression in the heart of both mice.

We next assessed the expression levels of *Col1a1* and *Col1a2*, which participate in cardiac fibrosis—they were significantly elevated in *Zip13*-KO mice before Dox administration (Fig 4E and 4F). In addition, the expression level of *MMP9*, a matrix proteinase regulating cardiovascular function, significantly decreased in WT mice injected with Dox to a level similar to that in *Zip13*-KO mice (Fig 4G), suggesting that expression level changes in *Col1a1*, *Col1a2*, and *MMP9* genes under ZIP13-deficient conditions may lead to the spontaneous arrhythmic features observed in *Zip13*-KO mice (Fig 3C, upper). We noted that the levels of *GM130*, a resident molecule in the Golgi apparatus, were significantly elevated in *Zip13*-KO mice after Dox injection (Fig 4H). In addition, the level of another Golgi apparatus resident protein, TGN46, was elevated in the heart tissue of *Zip13*-KO mice, as confirmed by IHC analysis (S1 Fig). ZIP13 localizes to the Golgi apparatus [6]. Hence, the loss of ZIP13 may result in impaired Golgi function in cardiac cells, leading to cardiovascular abnormalities.

Overall, our findings show that ZIP13 plays an important physiological role in the regulation of cardiovascular homeostasis, possibly by modulating genes associated with inflammation.

## Discussion

CVDs are amongst the major causes of death worldwide. The development of useful therapeutic agents and investigation of novel molecular mechanisms underlying CVDs are essential to overcome this global health problem [21]. There are several useful therapeutic drugs with various pharmacological effects that ameliorate symptoms of CVDs [21]. However, CVDs remain a significant global health concern, with high levels of associated mortality. Therefore, intensive research is required to develop novel drug candidates that target specific molecules and yield improved health outcomes. One potential target is the calcium signaling pathway mediated by L-type Ca^2+^ channels and the type 2 ryanodine receptor (RyR2). Cardiac muscle contraction is triggered mainly by intracellular calcium transient signaling via L-type Ca^2+^ channels and RyR2, which are localized in the sarcoplasmic reticulum (SR) [17]. These molecules transport Ca^2+^ into the cytosol, which acts as a second messenger to shift contractile myofibrils [22]. Dysregulation of calcium signaling induces the upregulation of reactive oxygen species (ROS), which increases oxidative stress in cardiomyocytes and aids in the pathogenesis of CVDs [23].

Considering this relationship, we assumed that zinc may be crucially involved in cardio-physiology and pathophysiology. Zinc is an essential mineral component involved in various physiological and cellular processes, including cardiovascular systems [24, 25]. Zinc deficiency causes numerous symptoms, such as hair loss, impaired inflammatory responses, taste disorders, growth retardation, and cardiac dysfunction [3]. Zinc homeostasis is regulated by two types of zinc transporter families, ZIP and ZnT, and amongst them, ZnT5 has been reported to be involved in heart function, and its deficiency causes osteopenia and sudden cardiac death [26], although information on the roles of zinc and its transporters is limited. Recently, several reports on the involvement of zinc ions and transporters in cardiovascular functions [14] have drawn attention to the involvement of ZIP13 in cardiovascular homeostasis. For example, genetic ZIP13-deficiency induced fragility of the aorta in mice [18]. ZIP13 is responsible for CaMKII activation, and ZIP13 downregulation exacerbates myocardial infarction by disrupting mitochondrial pathways [7]. In calcific aortic valve disease, which is the most prevalent heart valve disease, ZIP13 expression level is significantly elevated, and ZIP13 knockdown inhibits human valve interstitial cells in an *in vitro* calcification model [27]. Disruption of intracellular zinc content in cardiomyocytes, which is regulated by several zinc transporters including ZIP13, affects cardiovascular function [15, 16, 28]. These reports indicate that alterations in ZIP13 expression may occur due to cardiac stress, which may induce CVDs or promote their pathogenesis.

In fact, TPEN treatment, known to chelate intracellular zinc, induced profound morphological changes involved in the induction of cell toxicity, which were ameliorated by the addition of zinc, indicating that zinc is essential for cardiomyocytes to maintain physiological homeostasis (Fig 1A). We noted that *Zip13* expression level significantly decreased in cardiomyocytes treated with Dox (Fig 1B, right). Similarly, a decrease in *Zip13* expression level in the heart tissues of C57BL/6N mice intraperitoneally injected with Dox was observed (Fig 1C, right). Dox is known to have been used as an anticancer agent with specific adverse effects such as cardiotoxicity, which is observed not only in experimental conditions but also in clinical use [19]. Although Dox-induced cardiotoxicity is thought to be induced mainly by ROS production [29], these results indicate that changes in ZIP13 expression levels due to cardiac stress induced by Dox treatment could be problematic factors influencing the progression of CVDs.

To clarify the physiological involvement of ZIP13 in cardiac function, we investigated PNCs from *Zip13*-KO mice (KO-PNCs) that exhibited unusual morphology compared to control cells (Fig 2B, upper panel) and showed aberrant beating (Fig 2B, lower panel), indicating that ZIP13 is necessary for normal cardiomyocyte function. The GO term enrichment analysis based on the RNA-seq data of both WT- and KO-PNCs (Fig 2C and 2D) indicated that GO terms related to inflammation, immune responses, and cell adhesion molecules were significantly enriched (Fig 2E). This may be associated with previous observations that inflammatory responses are elevated in patients with CVDs and that cardiac fibrosis, a major CVD symptom, was partly induced by the impairment of the excessive production of matrix metalloproteinase (MMP) [30]. In addition to tissue culture–based analysis, we also conducted *in vivo* physiological examinations, such as telemetry analysis, which demonstrated that *Zip13*-KO mice indeed showed arrhythmia (Fig 3C and Table 1), reminiscent of the abnormal rhythm of the KO-PNCs (Fig 2). Two-way ANOVA revealed that both genotype and Dox injection independently influenced the impairment of cardiovascular function, and there was no interaction between genotype and Dox injection that influenced cardiac dysfunction (Table 2). These results indicate that ZIP13 is essential for maintaining physiological cardiac function.

We also performed gene expression profiling to confirm the role of ZIP13 in regulating gene expression. We developed a DOX-induced CVD model in which CVD markers such as *BNP* and *eNOS* (Fig 4A and 4B) were found to be increased. *Trp53*, which is known to exhibit protective effects against cardiotoxicity [20], was also significantly increased in the CVD model using WT and *Zip13*-KO mice (Fig 4C), as was the case for HDAC4 expression (Fig 4D). HDAC expression level increased in response to cardiotoxic stimuli, and this was correlated with CVD symptoms [31]. HDAC4 contains a zinc-binding domain in its structure [32]. Hence, the disruption of intracellular zinc homeostasis in *Zip13*-KO cells in the heart might affect the expression or function of HDACs, which may lead to the dysregulation of gene expression in the heart. Notably, the expression levels of extracellular matrix genes, including *Col1a1* and *Col1a2*, significantly increased, and the level of matrix proteinase MMP9 showed a decreasing trend in *Zip13*-KO mice (Fig 4E–4G). An increase in COL1A expression level in the heart is involved in the onset or progression of cardiac fibrosis; in addition, MMP9 regulates cardiac function, and MMP9 disruption results in cardiac failure [33]. ZIP13 is a key molecule for collagen-containing hard and connective tissue development, and loss of function of ZIP13 impairs the expression of these genes in mouse and humans [6]. Therefore, ZIP13 deficiency may cause abnormalities in cardiac stroma tissues, leading to arrhythmia, as observed in the telemetry ECG analysis.

In addition to these observations, we have noted that the level of GM130, which is expressed on the Golgi apparatus membrane, increases in response to Golgi stress [34] which is a newly proposed cellular response system [35]. Furthermore, the protein expression level of TGN46, another Golgi apparatus resident protein, also increased in the heart tissue of *Zip13*-KO mice (S1 Fig). This link between ZIP13 and Golgi-related proteins suggests the fundamental involvement of ZIP13 in regulating the functions of the Golgi apparatus. The relationship between the Golgi apparatus and cardiovascular function has been reported, revealing that Golgi stress disrupts intracellular Na^+^ levels and K^+^ and Ca^2+^ transport in cardiac myocytes [36]. ZIP13 is expressed in the Golgi apparatus, and *Zip13*-KO mice with Dox injection significantly upregulated the expression of GM130 (Fig 4H), which suggested that the lack of ZIP13 expression exacerbated Golgi stress, which may induce cardiomyocyte dysregulation.

Our findings were obtained from *in vitro* and *in vivo* mouse model experiments. Therefore, additional efforts are required to clarify the function of ZIP13 in human cardiac cardiovascular homeostasis. The results presented herein might be involved in case where clinical information stated that one female patient had a medical history of stroke and cerebral hemorrhage (this information was provided by personal communication of the two EDSSPD3 patients [6] and their primary doctors), as described in the case report section. Although there was no evidence identifying the major causes of stroke and cerebral hemorrhage in the patient at this moment, our data may provide important cues for regular health checks of the cardiovascular function of EDSSPD3 patients. The report that ZIP13 is involved in extracellular matrix production (and its loss of function may increase aortal fragility) may be of particular interest to related studies [18, 37]. These findings suggest that ZIP13 may play a role in attenuating inflammatory responses and fine-tuning the expression of cell adhesion molecules that regulate cardiovascular functions *in vivo*.

## Conclusions

ZIP13 is a critical regulator of signaling in the normal and diseased states of cardiac function. Our data emphasizes that the modulation of ZIP13 expression may exert profound effects on inflammatory signaling in response to endogenous and exogenous stimulation, which may provide new possibilities for developing therapeutic agents for CVD treatment in the future.

## Supporting information

S1 Fig The expression of TGN46 in heart tissues of *Zip13*-KO mice. Immunofluorescence staining of heart tissue sections using antibodies against TGN46 and laminin-2α in WT and *Zip13*-KO mice. Two representative images each were obtained from WT and *Zip13*-KO mice.
(TIF)

S1 Video Morphology and beating of PNCs derived from WT mice. (MP4)

S2 Video Morphology and beating of PNCs derived from *Zip13*-KO mice. (MP4)
