# Supplementary figures and images for: Role of Scl39a13/ZIP13 in cardiovascular homeostasis
Source: PLoS One. 2022 Oct 21;17(10):e0276452. doi: 10.1371/journal.pone.0276452 (PMC9586387; doi:10.1371/journal.pone.0276452)

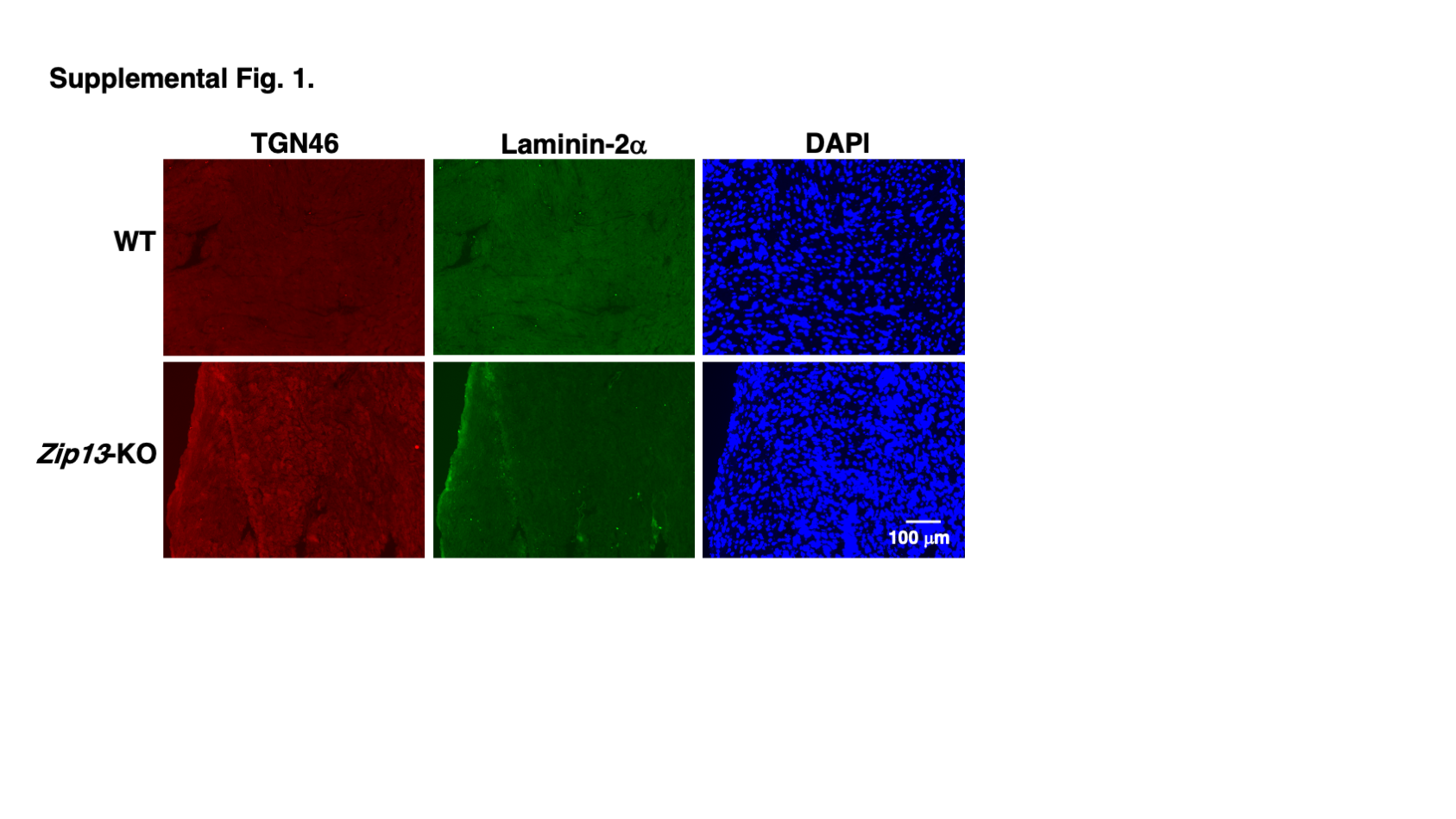

Supplement: S1 Fig — Immunofluorescence staining of heart tissue sections using antibodies against TGN46 and laminin-2α in WT and Zip13-KO mice. Two representative images each were obtained from WT and Zip13-KO mice. (TIF) [file pone.0276452.s001.tif]
